# The Effect of Force-Field Parameters on Cytochrome P450-Membrane Interactions: Structure and Dynamics

**DOI:** 10.1038/s41598-020-64129-7

**Published:** 2020-04-29

**Authors:** Ghulam Mustafa, Prajwal P. Nandekar, Goutam Mukherjee, Neil J. Bruce, Rebecca C. Wade

**Affiliations:** 1 Molecular and Cellular Modeling Group, Heidelberg Institute for Theoretical Studies (HITS), Heidelberg, Germany; 2 Zentrum für Molekulare Biologie der Universität Heidelberg, DKFZ-ZMBH Alliance, INF 282, 69120 Heidelberg, Germany; 3 Interdisciplinary Center for Scientific Computing (IWR), Heidelberg University, INF 368, 69120 Heidelberg, Germany; 4 Present Address: B-Zell-Immunologie (D130), German Cancer Research Center, Deutsches Krebsforschungszentrum (DKF), Im Neuenheimer Feld 280, 69120 Heidelberg, Germany; 5 Present Address: Schrodinger Inc. #147, 3rd Floor, Jawaharlal Nehru main road, Above State Bank of India, Channasandra, 5th Stage, RR Nagar, Bengaluru, 560098 India

**Keywords:** Biochemistry, Biophysics, Chemical biology, Computational biology and bioinformatics, Structural biology

## Abstract

The simulation of membrane proteins requires compatible protein and lipid force fields that reproduce the properties of both the protein and the lipid bilayer. Cytochrome P450 enzymes are bitopic membrane proteins with a transmembrane helical anchor and a large cytosolic globular domain that dips into the membrane. As such, they are representative and challenging examples of membrane proteins for simulations, displaying features of both peripheral and integral membrane proteins. We performed molecular dynamics simulations of three cytochrome P450 isoforms (2C9, 2C19 and 1A1) in a 2-oleoyl-1-palmitoyl-sn-glycerol-3-phosphocholine bilayer using two AMBER force field combinations: GAFF-LIPID with ff99SB for the protein, and LIPID14 with ff14SB for the protein. Comparison of the structural and dynamic properties of the proteins, the lipids and the protein-membrane interactions shows differing sensitivity of the cytochrome P450 isoforms to the choice of force field, with generally better agreement with experiment for the LIPID14 + ff14SB combination.

## Introduction

**Figure 1:**
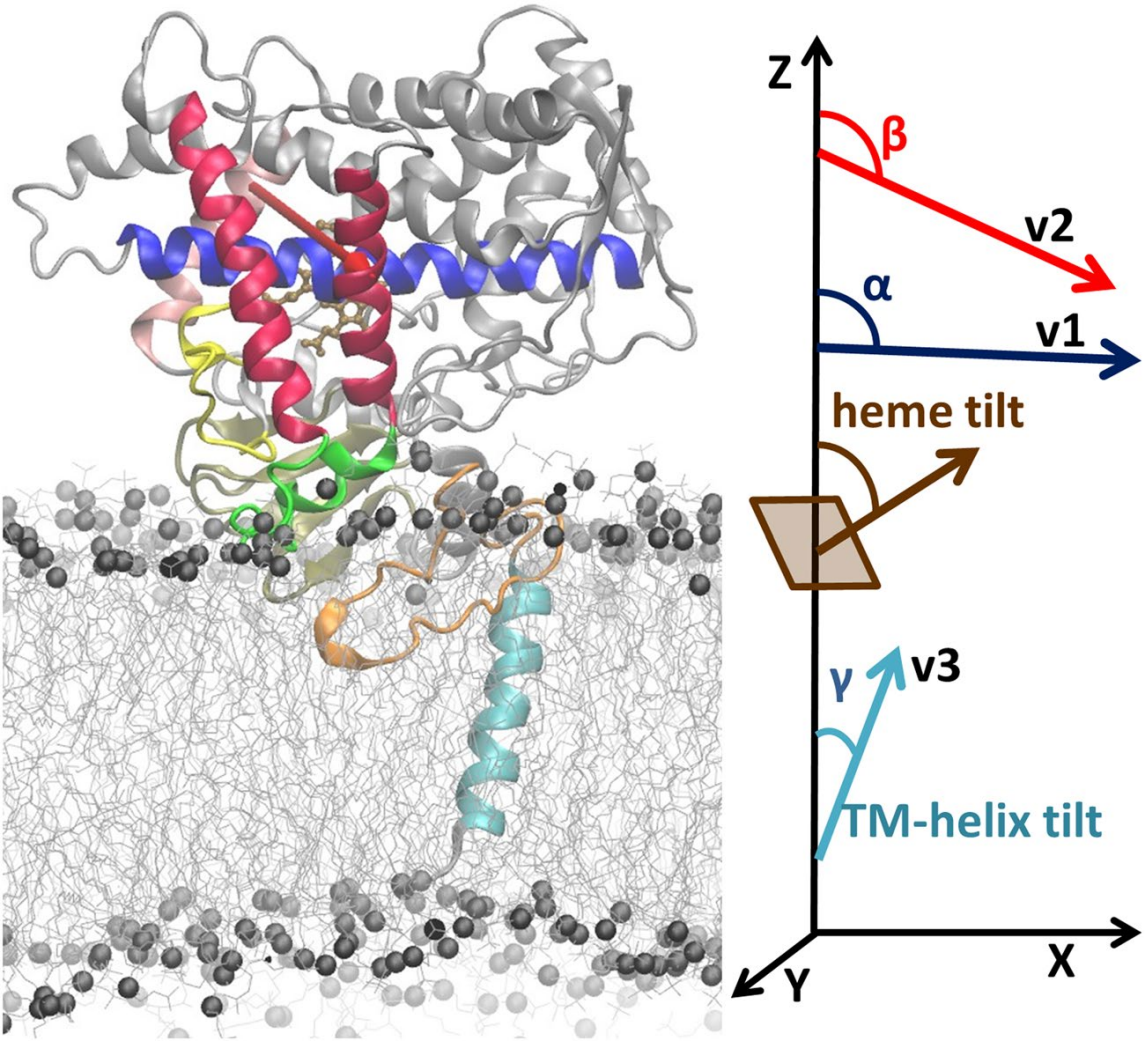
Model of CYP 2C9 (ribbon with heme in brown stick representation) in a POPC bilayer. Regions important for interactions with the membrane are colored: yellow: BC loop; dark red: F and G helices, green: FG loop containing F’ and G’ helices, pink: C helix, blue: I helix, magenta: β1 sheet, orange: linker, cyan: TM-helix. Gray spheres represent the phosphorous atoms of the POPC head groups. The angles and vectors (v1 along the I-helix, v2 shown by red arrow from the C to the F helix, v3 along the TM-helix) that characterize the orientation of the protein in the membrane are shown on the right and their definitions are given in the Methods. The snapshot is obtained from the final frame of the simulation with LIPID14 + ff14SB (see below). The image was generated using VMD 1.9 (www.ks.uiuc.edu/Research/vmd/)^20^.

Molecular dynamics (MD) simulation provides a powerful approach to obtain detailed insights into the structure and dynamics of complex biomolecular assemblies, such as protein-membrane systems. Membrane proteins have many important biological roles, e.g. as receptors, channels, transporters and enzymes, and they are targets for about 50% of all marketed drugs^1^. Membrane proteins have a range of transmembrane topologies. The largest class has a single α-helical transmembrane (TM) helix^2,3^. Cytochrome P450 (CYP) enzymes belong to this class and they have a TM helix anchor that spans the membrane bilayer and is connected by a flexible linker to a large cytosolic globular domain that dips into the membrane (Fig. 1**)**. Thus, CYP enzymes embody the key features of membrane proteins and thereby provide excellent systems for testing force fields (ff) for the simulation of membrane proteins. Moreover, CYPs are key enzymes in a number of important cellular functions, including the metabolism of endogenous and xenobiotic compounds and steroidogenesis^4^. There is consequently a growing interest in studying the structure-function relationships of CYPs, and MD simulations can be expected to play an increasing role in these studies. Experiments with CYPs in various environments from detergents to micelles, bicelles and Nanodiscs have provided information on how CYPs interact with membranes^5–8^. However, to provide a complete structural and dynamic picture, models of CYPs in membranes have been built and simulated and these studies have been conducted using a number of different force fields, see e.g. ^9–15^. Here, we use a multiresolution modeling and simulation procedure that we have previously developed for predicting CYP-membrane interactions using coarse-grained (CG) and atomic-detail (AA) MD simulations and that gave results consistent with available experimental data^16–19^.

MD simulations have been widely used to simulate phospholipid bilayers and systems with transmembrane proteins and peptides. Different ffs for simulations of phospholipid bilayers have been compared in a number of studies^21–24^. Similarly, protein ffs for simulating proteins in aqueous solution have been evaluated^25–28^. Only recently has the compatibility of protein and lipid force fields, including the AMBER ff14SB/Slipids and AMBER ff14SB/Lipid14 combinations, been critically evaluated^29^. However, further analysis of the compatibility of the lipid ffs with protein ffs, which is crucial for studies of protein-membrane interactions, remains lacking. Here, we address this need by performing simulations of three CYP isoforms, CYP 2C9, CYP 2C19 and CYP 1A1, in a 2-oleoyl-1-palmitoyl-sn-glycerol-3-phosphocholine (POPC) membrane using two AMBER family ff combinations: GAFF-LIPID with AMBER ff99SB (GAFF-LIPID + ff99SB), and LIPID14 with AMBER ff14SB (LIPID14 + ff14SB). In both cases, the TIP3P model^30,31^ was used for water. Both the older GAFF-LIPID + ff99SB combination and the newer LIPID14 + ff14SB combination have been used in prior simulations of cytochrome P450 enzymes and other membrane proteins, see for example refs. ^14–16,29,32^. Simulations were performed for a POPC bilayer because POPC is a major component of the endoplasmic reticulum (ER) membrane^33^, in which these CYPs are embedded *in vivo*^34^, and because *in vitro* experiments have been performed for CYPs in POPC Nanodiscs^35^. We compared the structural, dynamic and interaction properties of the simulated systems.

## Results and Discussion

We first built CG models of the CYP-membrane systems with different conformations of the flexible linker. For each system, five independent CG simulations of 6–10 microseconds duration were run to efficiently sample configurational space, and these resulted in a converged orientation of the globular domain in the bilayer in times of up to 4 microseconds, see Supplementary Fig. S1. A representative structure of each CYP-membrane system from the CG simulations was then converted to AA representation and relaxed and refined by running AA MD simulations with the two ffs. Because of the prior sampling in CG simulations, reasonable convergence of the properties of the systems was achieved in most of the AA MD simulations within times of 70–220 ns. However, we noted that in some of the simulations with the GAFF-LIPID + ff99SB combination, the systems showed structural divergence after simulation times of about 50 ns and we therefore ran two replicas of the GAFF-LIPID + ff99SB simulations for each of the three protein-membrane systems. An additional replica simulation was also performed for CYP 1A1 with LIPID14 + ff14SB.

### Protein-membrane interactions

Parameters^18^ computed to characterize the orientation and position of the CYP globular domain with respect to the membrane during the AA MD simulations are given in Table 1 and the coordinates of final snapshots from the simulations are provided in six PDB files as Supplementary Data. The computed angles are indicated in Fig. 1 and defined in Methods. The heme tilt angles are mostly lower in the simulations with GAFF-LIPID + ff99SB than with LIPID14 + ff14SB. Although the heme tilt angle has not been measured for any of the three CYP isoforms studied here, the values of the heme tilt angle obtained in the simulations with LIPID14 + ff14SB for CYP 1A1 and CYP 2C19 are similar to the range (57–62°) that has been measured for other CYP isoforms in a POPC Nanodisc by linear dichroism^35^. The α and β angles, which define the orientation of the CYP globular domain with respect to the membrane^16^, showed correspondingly similar trends for the two ffs, with the greatest differences, of about 25°, for CYP 2C19, and with the differences partially due to distortion of the helices used to define the angles in the GAFF-LIPID + ff99SB simulations (see below). The TM-helix tilt angles observed for the three CYPs in the simulations are quite similar to the value of about 17° observed for CYP 2B4 in DLPC/DHPC bicelles^36^.

**Table 1:** Parameters characterizing the orientation and depth of insertion of the CYP globular domain in the membrane bilayer, as well as the orientation of the TM-helix in the bilayer, in AA MD simulations for three CYP isoforms with GAFF-LIPID + ff99SB and with LIPID14 + ff14SB.

CYP Isoforms	CYP 2C9		CYP 2C19		CYP 1A1	
Force fields	GAFF-LIPID +ff99SB	LIPID14 + ff14SB	GAFF-LIPID +ff99SB	LIPID14 + ff14SB	GAFF-LIPID +ff99SB	LIPID14 + ff14SB
Computed Parameters						
Protein CoM to membrane CoM distance (Å)	40.3 ± 0.7 *38.6 ± 0.9*	45.5 ± 1.5	43.2 ± 0.9 *46.2 ± 1.7*	46.1 ± 2.6	36.0 ± 0.7 *40.2 ± 1.2*	40.6 ± 1.5 *42.3 ± 1.6*
Heme tilt angle (^o^)	33.1 ± 3.7 *28.6 ± 4.4*	43.2 ± 4.8	42.1 ± 3.7 *47.2 ± 4.5*	60.5 ± 4.5	66.1 ± 2.9 *51.4 ± 4.5*	54.8 ± 6.1 *53.5 ± 4.2*
α angle (^o^)	92.1 ± 2.4 *79.4 ± 3.4*	74.8 ± 4.3	76.8 ± 2.9 81.4 ± 4.4	106.3 ± 4.2	95.8 ± 1.6 96.4 ± 4.6	109.7 ± 4.6 *85.0 ± 5.6*
β angle (^o^)	113.2 ± 2.5 *112.2 ± 2.8*	119.9 ± 4.5	122.7 ± 3.4 *129.2 ± 3.8*	148.6 ± 5.1	145.5 ± 1.7 *135.8 ± 4.9*	149.0 ± 2.9 *121.2 ± 4.8*
TM-helix tilt angle (^o^)	17.9 ± 3.8 *15.6 ± 3.3*	11.9 ± 5.3	7.4 ± 4.1 *14.4 ± 3.7*	25.4 ± 7.8	28.4 ± 2.6 *22.6 ± 4.4*	23.4 ± 4.3 *21.9 ± 5.3*

Means and standard deviations were computed over the last 50 ns of each MD production trajectory. For cases where two replica simulations were performed, the values for the longer simulation are given followed by those of the shorter simulation in italic. Trajectory lengths are given in Table 2.

**Figure 2:**
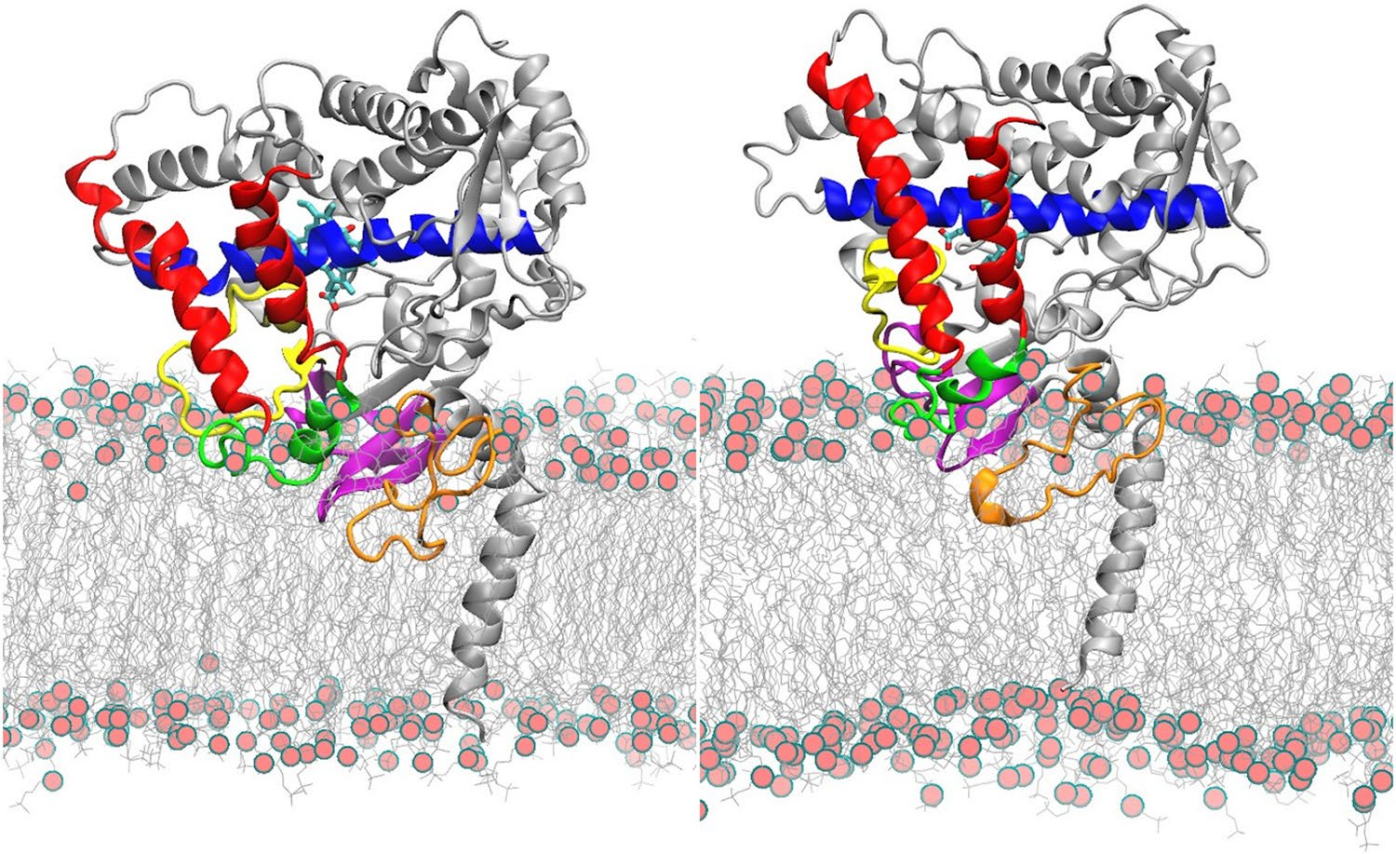
Comparison of the final orientations of CYP2C9 in the POPC bilayer in simulations of the apoprotein with GAFF-LIPID + ff99SB after 76 ns (left) and LIPID14 + ff14SB after 216.9 ns (right). The globular domain is less immersed in the membrane in the simulations with LIPID14 + ff14SB than with GAFF-LIPID + ff99SB. The interactions and dynamics of regions lining the substrate access routes to the active site from the membrane, especially the FG loop, differ, see Table 1 and Fig. 3. Color scheme: The protein is shown in cartoon representation as follows: yellow: BC loop; dark red: F and G helices, green: FG loop containing F’ and G’ helices, blue: I helix, magenta: β1 sheet, orange: linker. The heme is shown in stick representation colored by atom type with cyan carbon atoms. Red spheres represent the phosphorous atoms of the head groups of the POPC lipids shown in grey line representation. The image was generated using VMD 1.9 (www.ks.uiuc.edu/Research/vmd/)^20^.

The axial distance between the center of mass (CoM) of the globular domain and the CoM of the membrane, characterizing the depth of insertion of the globular domain in the membrane, was generally longer for LIPID14 + ff14SB than for GAFF-LIPID + ff99SB, as illustrated for CYP 2C9 in Fig. 2. There was thus less immersion of the protein in the membrane and therefore, a higher degree of freedom for motion of the globular domain for LIPID14 + ff14SB. The shallower insertion of the proteins in the bilayer with LIPID14 + ff14SB than with GAFF-LIPID + ff99SB may be due to differences in polar interactions and the structure and mobility of the lipid bilayer, see below.

### Structural and dynamic properties of the proteins

For all three proteins with LIPID14 + ff14SB, the Cα atom root mean squared deviation (RMSD) of the globular domain with respect to the energy minimized structure rose to about 2–2.5 Å and then remained stable during the MD simulations, indicating the overall stability of the CYP globular domain during MD simulations (Supplementary Fig. S2**)**. With GAFF-LIPID + ff99SB the RMSD evolved to 2.5–3.5 Å during the simulations, with the RMSD mostly showing a tendency to rise quicker with simulated time than for LIPID14 + ff14SB. The trends in RMSD overall indicate somewhat greater preservation of the crystallographic protein structures in the simulations with LIPID14 + ff14SB than with GAFF-LIPID + ff99SB.

**Figure 3:**
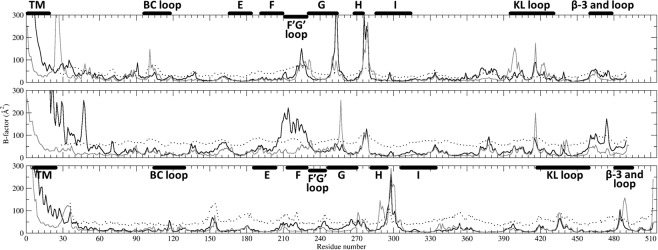
Variation of the average B-factor values (8π^2^RMSF^2^/3) of protein backbone Cα atoms along the sequences of CYP 2C9 (top), CYP 2C19 (middle) and CYP 1A1 (bottom). Values computed from AA MD simulations with LIPID14 + ff14SB (black lines) and GAFF-LIPID + ff99SB (gray lines) are compared with crystallographic B-factors (black dotted lines). For systems for which replica simulations were run, the values are given for the longer simulation. The locations of secondary structure elements are shown by bars. For CYP 2C9 and CYP 2C19, the residue numbers for the secondary structure are the same. The plots were generated using Xmgrace (plasma-gate.weizmann.ac.il/Grace/)^38^.

The trends in variation of the computed B-factors along the sequences overall correspond to those for the crystallographic B-factors (Fig. 3). There are however some notable differences between the proteins and between the two sets of ff parameters. The regions of the proteins in contact with the membrane, such as the F’G’ region, show higher mobility in simulations with LIPID14 + ff14SB than with GAFF-LIPID + ff99SB overall (Fig. 3). This observation is consistent with the higher mobility of the phospholipids^37^ and the reduced depth of insertion of the globular domain in the membrane with LIPID14 + ff14SB than with GAFF-LIPID + ff99SB. In all proteins, the linker, connecting the TM-helix with the globular domain, is more flexible in the simulations with LIPID14 + ff14SB than GAFF-LIPID + ff99SB, consistent with the greater fluctuation of the TM-helix angle with LIPID14 + ff14SB (Table 1).

In CYP 1A1 using LIPID14 + ff14SB, somewhat higher fluctuations were seen in the EF loop, F helix and FG loop but the secondary structure showed similar behavior for both ffs (Supplementary Fig. S5). For CYP 2C9 with GAFF-LIPID + ff99SB, however, secondary structure distortion or bending of helices was observed in the F’, G’ and G helices, which line the proposed substrate access route and the active site, along with some unwinding of the central I helix (Supplementary Fig. S3). In addition, with GAFF-LIPID + ff99SB, CYP 2C9 showed higher mobility in some regions (especially the BC and KL loops) than in the crystal structure and in the simulations with LIPID14 + ff14SB (Fig. 3). The increased flexibility in the BC loop resulted in formation of a wide tunnel (tunnel 2d/f)^39^ leading to the binding pocket with increased interactions of the BC loop with the membrane headgroups. In simulations of CYP 2C19, distortion in the F helix residues 205–208 close to the F’ helix and unwinding in the I helix was also observed with GAFF-LIPID + ff99SB (Supplementary Fig. S4). However, the particularly high fluctuations in the F’G’ helices in CYP 2C19 for LIPID14 + ff14SB (Fig. 3) could be partly due to unwinding of the F’G’ helices (Supplementary Fig. S4). In simulations of ligand-bound CYP2C9 and CYP 2C19 in a POPC bilayer with LIPID14 + ff14SB (Mustafa *et al*.^40^), we observed that a few amino acid substitutions in the F’G’ and β1-1 and 1-2 regions resulted in different contacts with the lipid bilayer and differing orientations of the CYP globular domain in the bilayer. In the present simulations of apo-CYP 2C19 with LIPID14 + ff14SB, we observed that conformational changes in the linker resulted in the re-orientation of charged residues in the linker to interact with the polar headgroups of the membrane. This re-orientation may contribute to the considerable difference in orientation of the globular domain of CYP 2C19 in the membrane for the two ffs.

### Structural and dynamic properties of the phospholipid bilayer

**Figure 4:**
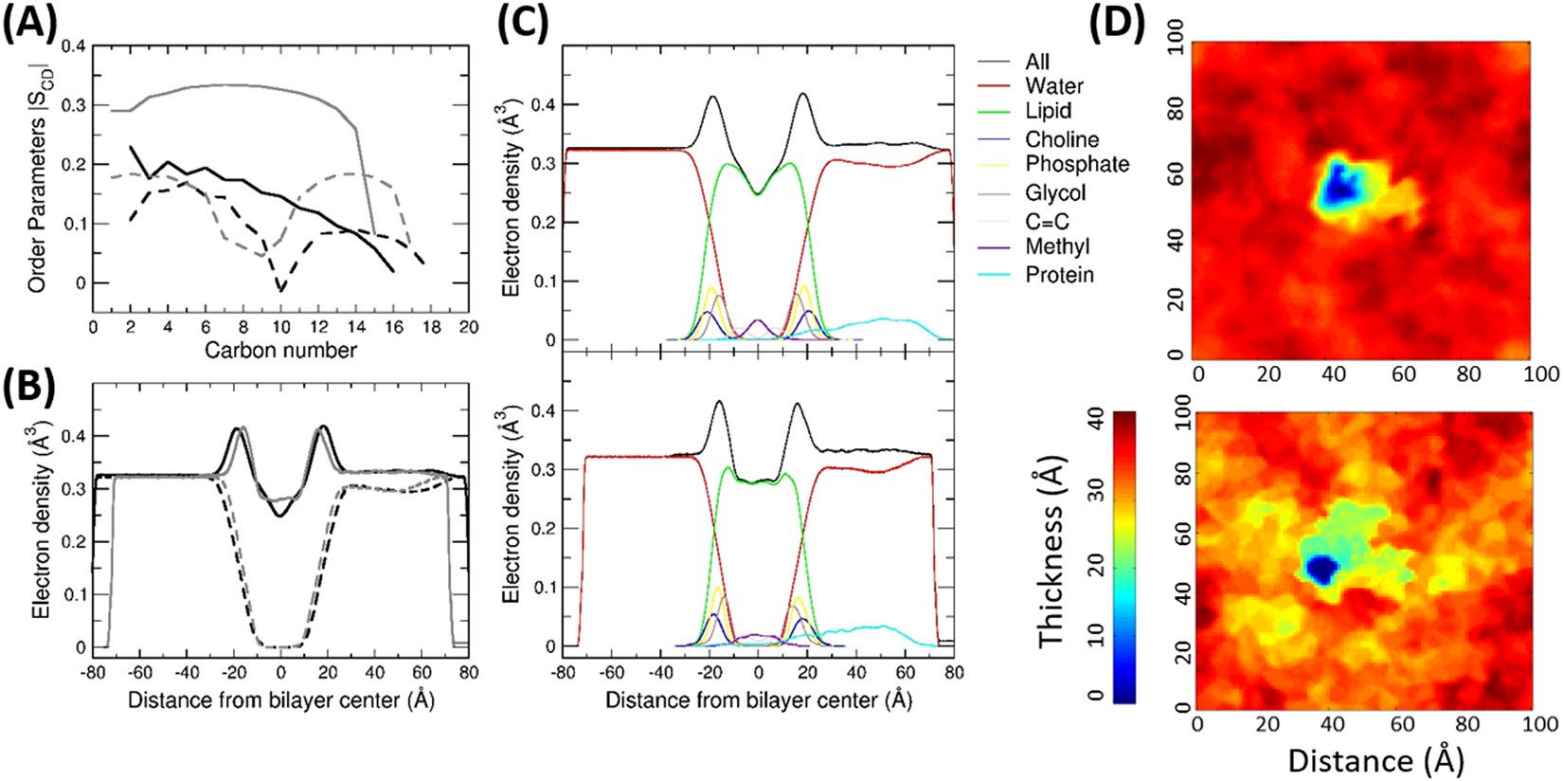
Comparison of the structural and dynamic properties of the phospholipid bilayer in simulations of the CYP 2C9-POPC membrane system conducted with the LIPID14 + ff14SB and the GAFF-LIPID + ff99SB parameters. Computed time averaged C-H (deuterium) order parameters for the Sn1 (solid lines) and Sn2 (dashed lines) chains (**a**) and electron density profiles (**b**) for simulations with LIPID14 + ff14SB (black) and GAFF-LIPID + ff99SB (grey) parameters. The electron density profiles computed for the last 50 ns of the simulations are shown for the protein+phospholipid system (upper lines) and for the water molecules (lower lines). The profiles peak at 0.43 e/Å^3^ for the head groups and drop below 0.3 e/Å^3^ for the methylene groups in the tail region^48^. The thickness of the bilayer is lower for GAFF-LIPID + ff99SB and the electron density is less depleted in the middle of the bilayer. The asymmetry in the electron density distribution around the bilayer center is due to the interaction of the protein globular domain on the positive side of the z-axis of the bilayer. The decomposition of the electron density profile into different segments of the membrane bilayer, water and protein is shown in (**c**) and the variation in the bilayer thickness over the simulated bilayer (**d**) for LIPID14 + ff14SB (upper panels) and GAFF-LIPID + ff99SB (lower panels). A similar shape of the trough in the profile is seen in all systems simulated with LIPID14 + ff14SB, whereas for GAFF-LIPID + ff99SB, a broader trough is visible. The plots in Fig. 4A–C were generated using Xmgrace (plasma-gate.weizmann.ac.il/Grace/)^38^. Fig. 4D was generated using g_lomepro v1.0.2 (www3.mpibpc.mpg.de/groups/de_groot/g_lomepro.html)^50^.

In a recent evaluation of AMBER ffs for lipids, the LIPID14 ff showed improved structural properties over the LIPID11/GAFF ff without the need to apply a constant surface tension in MD simulations of bilayers^37^. However, proteins can influence membrane properties as much as membranes can shape the structure and function of proteins. Therefore, the structure and dynamics of the membrane in the presence of protein were examined by computing the membrane thickness, the surface area occupied by each lipid (area per lipid (APL)), lipid order parameters, and the electron density profile across the membrane (Table 2, Fig. 4 and Supplementary Fig. S6), and comparing with experimental structural data on lipid bilayers^33,41–43^.

**Table 2:** Structural properties of the phospholipid bilayer computed from AA MD simulations of the three CYP isoforms in a POPC bilayer with two sets of ff parameters.

	Time(ns)	Cell Dimensions^a^ (Å)		Number of lipids^a^		Average APL (Å^2^)^b^		
		X	Y	Boundary^c^	Non-Boundary^c^	Boundary^c^	Non-Boundary^c^	All
**CYP 2C9**								
Start frame	0.0	142.4	138.1	49	545	51.7 ± 14.1	64.9 ± 15.8	63.8 ± 16.0
GAFF-LIPID + ff99SB	139.4 *76.0*	148.2 *150.6*	152.2 *150.1*	75 78	519 516	55.9 ± 2.0 *55.2* ± *2.1*	73.7 ± 0.6 *72.2* ± *1.5*	71.4 ± 0.6 69*.9* ± *1.5*
LIPID14 + ff14SB	216.9	151.7	130.9	51	543	48.1 ± 2.1	66.1 ± 0.7	64.4 ± 0.7
**CYP 2C19**								
Start frame	0.0	142.4	138.1	54	540	44.3 ± 22.2	65.9 ± 12.7	64.0 ± 15.1
GAFF-LIPID + ff99SB	136.7 *69.7*	150.2 *142.1*	143.0 *144.5*	75 *69*	519 525	55.0 ± 1.8 *56.7* ± *1.8*	71.3 ± 0.5 *68.3* ± *0.5*	69.4 ± 0.5 *67.0* ± *0.5*
LIPID14 + ff14SB	108.4	141.3	141.5	63	531	49.3 ± 3.0	66.2 ± 0.6	64.4 ± 0.7
**CYP 1A1**								
Start frame	0.0	142.4	138.1	47	547	56.7 ± 18.5	65.1 ± 13.3	64.5 ± 13.9
GAFF-LIPID + ff99SB	211.2 *185.0*	144.9 *152.4*	135.8 *137.1*	98 89	496 505	47.5 ± 1.1 *56.2* ± *2.7*	62.4 ± 0.7 *65.8* ± *0.9*	59.8 ± 0.7 *64.3* ± *1.2*
LIPID14 + ff14SB	225 *217.0*	132.4 *133.7*	152.2 *151.3*	79 76	515 518	44.1 ± 2.4 *48*.*6* ± *2.0*	66.3 ± 0.6 *66.6* ± *0.7*	63.4 ± 0.6 *64.5* ± *0.5*

For cases where two replica simulations were performed, the values for the longer simulation are given followed by those of the shorter simulation in italic.

^a^Cell dimensions and numbers of lipids are given for the initial and final frames of the production runs at the times indicated and show the evolution of the simulation box with time.

^b^Average values and standard deviations of the area per lipid (APL) are given. For the initial frame, averaging is over all lipids in the specified region. For the final frames, averaging is over snapshots taken at 0.2–1 ns intervals over the last 50 ns of each simulation and over all lipid molecules in the specified region for each snapshot. For comparison, previously reported values for pure POPC bilayers from MD simulation^37^ are 65.6 Å^2^ and from experiments are 64.3, 68.3^33,41^, and 66.0^44^ Å^2^.

^c^In any given trajectory snapshot, lipid molecules within 5 Å of the protein atoms were designated as boundary lipids and those beyond were designated as non-boundary lipids.

The average APL of the non-boundary (bulk) lipids in all the simulations with LIPID14 + ff14SB was consistent with experiments for POPC bilayers^33,41,44^. The APL of the boundary lipids next to the protein was lower due to protein interactions. However, in the simulations with GAFF-LIPID + ff99SB, the box dimensions and number of boundary lipids increased along with the APL of the non-boundary lipids (Fig. S6). Visual inspection of the simulations of CYP 2C9 with GAFF-LIPID + ff99SB showed that the lipids became highly ordered and that empty voids between the lipid molecules contributed to the increased average APL. The increase in the number of boundary lipids corresponds with increased protein-membrane contacts and the deeper insertion of the protein in the membrane with GAFF-LIPID + ff99SB. Order parameters for the lipid Sn1 and Sn2 chains computed from the CYP 2C9 simulations with LIPID14 + ff14SB (Fig. 4) match well with order parameters for a pure POPC bilayer determined experimentally^45,46^ and from simulation results^37^. The order parameters computed from the simulations with GAFF-LIPID + ff99SB are all higher, with particularly high values for the saturated Sn1 chain, indicating a rigid gel-like configuration of the lipids. Higher order parameters were also computed from an earlier simulation of a pure POPC bilayer with GAFF-LIPID^46^.

Along with the thickness of the bilayer, defined as the distance between two headgroup peaks^45,47^, the density profile also gives information about the hydration shell and insertion of water molecules in the lipid membrane^47,48^ (Fig. 4**)**. The membrane thickness was inversely related to the APL. The electron density profiles computed from the simulations with the two parameter sets differ in shape. All simulations with LIPID14 + ff14SB show a similar bilayer shape. The calculated thickness of the bilayer for the CYP 2C9 system simulated with LIPID14 + ff14SB was 37.1 Å, which matches well with the value previously reported for pure POPC bilayers from MD simulations with LIPID14^37^ (36.9 ± 0.6 Å) and from experiments^41^ (37 Å). The thickness of the bilayer for the CYP 2C9 system simulated with the GAFF-LIPID + ff99SB was lower, 32.2 Å, and more varied over the bilayer (Fig. 4**, right**) and the electron density profile (Fig. 4**, middle**) showed a broad trough in the tail region, indicating that an interdigitated bilayer was formed. A similar effect was observed in small- and wide-angle X-ray scattering (SWAXS) experiments on the multifunctional human peptide LL-37 which causes membrane disruption^49^. The decomposition of the electron density profile into its components (Fig. 4**, middle panels**) shows, as expected from the lower CoM-CoM distance between the protein globular domain and the membrane, that the protein penetrates further into the membrane region in the simulations with GAFF-LIPID + ff99SB.

Overall, in the simulations with LIPID14 + ff14SB, the non-boundary phospholipids show behavior consistent with that known for bulk POPC whereas these phospholipids tend to deviate from this bulk behavior during the simulations with GAFF-LIPID + ff99SB. Furthermore, the use of GAFF-LIPID + ff99SB results in more phospholipid molecules interacting closely with the protein.

## Conclusions

We compared the results of using two different AMBER ff combinations to simulate CYP-POPC bilayer systems. Atomic-detail MD simulations of up to about 200 ns duration were run after obtaining systems with converged arrangements of the proteins immersed in the bilayer from a set of independent coarse-grained simulations. While longer AA MD simulations might allow investigation of slow transitions in the CYP-POPC systems, the AA MD simulations run (including replicas) were of sufficient length to examine protein-membrane interactions and to reveal differences in the systems simulated with the two AMBER ff combinations. Mostly, the results indicate that the combination of LIPID14 for the membrane with ff14SB for the protein can be preferred over GAFF-LIPID + ff99SB to study such protein-membrane interactions. Moreover, the results with the LIPID14 + ff14SB combination show similar or better agreement with experiment compared to results obtained for CYP-membrane systems with other ffs, such as CHARMm and GROMOS^9,11^. The LIPID14 + ff14SB combination reproduces the structural and dynamic properties of the POPC phospholipid bilayer better than GAFF-LIPID + ff99SB. We found it necessary to use a smaller timestep of 1.5 fs with the GAFF-LIPID + ff99SB combination in order to run simulations of over 100 ns while maintaining a reasonable bilayer structure. The GAFF-LIPID ff requires application of a surface tension to maintain the membrane bilayer, whereas with LIPID14, anisotropic pressure coupling can be used. The effect of the ff on protein structure and dynamics and protein-membrane interactions was subtler. For CYP 1A1, the protein properties showed only small variations with ff, whereas larger effects were observed for CYP 2C9 and CYP 2C19, indicating that the protein stability in the membrane is dependent on specific sequence differences between the isoforms. These differences in stability may be related to the finding that different CYPs have preferences for different regions of membranes with some preferring to be in lipid rafts and some outside and CYP1A1 localizing in disordered regions of the ER membrane^51–53^.

Consistent with the improved backbone and sidechain parameters in ff14SB^54^, the protein secondary structure tended to be more stable in simulations with LIPID14 + ff14SB than with GAFF-LIPID + ff99SB, despite higher fluctuations in the parts of the protein interacting with the membrane due to the greater lipid mobility.

In this case study, we have compared the performance of two ff combinations for three proteins with the same CYP fold in a phospholipid bilayer composed of one type of lipid (POPC). Simulations of other CYPs in different lipid membranes or mixed composition membranes, for example with the addition or cholesterol or representing the ER composition, indicate that the protein dynamics and binding properties may be different from in a POPC membrane^54,55^. Furthermore, CYP-membrane interactions may be influenced by the binding of their redox partners, which are themselves membrane bound proteins with a transmembrane helical anchor^56^. Therefore, for a complete picture of the performance of the ff combinations studied here, further systematic studies of ff effects would be required for other proteins, for other phospholipids and for more realistic models of biological membranes consisting of a mixture of different constituents. On the other hand, the CYP-membrane systems studied here could provide useful test systems for the evaluation of further ffs and ff combinations.

## Computational Methods

Models of the full length CYP proteins in a POPC bilayer were built and simulated using MARTINI^57^ CG models and AA models as described previously^7,9^. Simulations were based on crystal structures of the globular domains of CYP 2C9 (PDB ID 1R9O;^59^ resolution 2.0 Å), CYP 2C19 (4GQS;^60^ resolution 2.87 Å) and CYP 1A1 (4I8V;^58^ resolution 2.6 Å). For AA MD simulations, either the AMBER ff14SB^61^ parameters were used for the protein with the LIPID14 parameters for the POPC lipids^37^ or the AMBER ff99SB parameters were used for the protein and the GAFF parameters for the lipid^46^. Simulations were carried out with NAMD 2.10^62^ in a periodic rectangular box with the TIP3P^30,31^ water model and with Na^+^ and Cl^-^ ions^63^ added to neutralize the system and maintain the ionic concentration at 150 mM. We ran AA MD simulations with the recommended pressure control for the respective ffs. The following sections provide the methodological details.

### Preparation of full-length CYP 2C9, CYP 2C19 and CYP 1A1

The following protein sequences and crystal structures from the RCSB-PDB (www.rcsb.org) were used: CYP 2C9 (Uniprot id P11712): PDB ID 1R9O^59^, resolution 2.0 Å, lacking N-terminal residues 1–25 and the F’G’ region (residues 214–220); CYP 2C19 (Uniprot id P33261): PDB ID 4GQS^60^, resolution 2.87 Å, lacking N-terminal residues 1–28; and CYP 1A1 (Uniprot id P04798): PDB ID 4I8V^58^, resolution 2.6 Å, lacking N-terminal residues 1–35. CYP 2C9 and CYP 2C19 have 91.2% sequence identity. As the missing residues in the linker and F’G’ region of CYP 2C9 are similar to those in CYP 2C19, the crystal structure of CYP 2C19 was used as a template for modeling these missing residues in CYP 2C9. The TM-helix (residues 3–21 for CYP 2C9, residues 2–23 for CYP 2C19 and residues 6–26 for CYP 1A1) and missing linker (residues 22–25 for CYP 2C9, residues 24–25 for CYP 2C19 and residues 27–36 for CYP 1A1) were modeled with a similar procedure to that used by Mustafa *et al*.^18^. The final models consisted of the crystal structure of the globular domain with modeled missing regions. Various starting orientations of the CYP globular domains above a POPC bilayer were generated by changing the dihedral angles in the flexible linker regions before conversion to the coarse-grained (CG) MARTINI representation. Subsequently, 5 independent CG MD simulations were performed using the GROMACS 5.0.4 software (www.gromacs.org) of each of CYP 2C9, CYP 2C19 and CYP 1A1 in a POPC bilayer for 6–10 µs each. During the CG MD simulations, the secondary and tertiary structure was maintained by elastic network restraints with an elastic force constant of 500 kJ/mol/nm^2^ and a distance cutoff range of 5–9 Å except in the flexible linker region (residues 22–36 for CYP 2C9, residues 26–38 for CYP 2C19, and residues 27–36 for CYP 1A1). The secondary structure information was provided for this purpose in a DSSP file obtained from the DSSP server^64^. (http://www.cmbi.ru.nl/dssp.html).

### Convergence of coarse-grained simulations and back conversion

The CG MD simulations were considered converged when no significant changes were observed in the orientation of the CYP globular domain with respect to the membrane as described previously^16–18^. The orientations were quantified by calculating the angles (α and β) and distances (center of mass (CoM) of the globular domain (residue 50–490 for CYP 2C9 and CYP 2C19 and residue 51 to 511 for CYP 1A1) or the FG loop from the CoM of the POPC membrane), defining the positioning of the globular domain above the lipid membrane. The interactions of the TM-helix in the membrane were quantified by computing the TM-helix tilt angle. Convergence plots are shown for CYP 1A1 in Fig. S1.

Representative frames of the converged orientations of each CYP isoform were selected for back-conversion to an all-atom (AA) model. The representative frame was chosen to have angle and distance values within 1% of the mean value for the saved snapshots of a particular orientation of each CYP. The back-conversion of the POPC bilayer was performed as described in Cojocaru *et al*.^16^, whereas the protein back-conversion was done using the scripts backward.py and initram.sh, available at the MARTINI website (cgmartini.nl)^65^. Due to the lack of the heme cofactor in the CG model, conformational changes in the side chains of heme-binding pocket residues were observed. Therefore, the globular domain from the crystal structure, including the heme-cofactor, was superimposed on the back-mapped structure and used in subsequent AA simulations. The TM-helix and the flexible linker region obtained from the back-conversion procedure were then connected to the globular domain, resulting in a full-length AA model, which was inserted into an AA model of the POPC bilayer to obtain a complete CYP-membrane complex.

### All-atom (AA) molecular dynamics simulations

AA MD simulations were performed for each CYP with two sets of force field parameters. Either the AMBER ff14SB^61^ parameters were used for the protein with the LIPID14 parameters for the POPC lipids^37^ or the AMBER ff99SB parameters were used for the protein and the GAFF force-field for the lipid^46^. Comparisons were made with our previous simulations of CYP 2C9 in a membrane^16^. The heme parameters were provided by D. Harris with the partial atomic charges derived from DFT calculations^66^. Simulations were carried out in a periodic rectangular box. Na^+^ and Cl^-^ ions were added to neutralize the protein and to maintain the ionic concentration at 150 mM and the TIP3P model^30,31^ was used for the water molecules. The same procedure for AA MD simulation was used as described in detail by Cojocaru *et al*^16^. and all simulations were carried out with NAMD 2.10^62^.

The simulation protocol began with energy minimization with a force constant on non-hydrogen atoms of protein and lipid residues that gradually decreased from 1000 to 0 kcal/mol.Å^2^, as described by Cojocaru *et al*^16^. The energy minimized systems were then equilibrated for 1.5 ns with a timestep of 1 fs maintaining constant surface area and pressure at a temperature of 310 K (in the NPAT ensemble), with a gradual decrease in harmonic restraints on non-hydrogen atoms of protein and lipid residues from 100 to 0 kcal/mol.Å^2^. Then the systems were equilibrated for 5 ns without any constraints in the NPAT ensemble. Afterwards, the production simulations were run in periodic boundary conditions. One simulation was run for each system using the LIPID14 + ff14SB combination and an additional replica simulation was run with this ff for the CYP 1A1 system. Two replica simulations (starting with different velocity assignments) were run with the GAFF + ff99SB combination due to the generally lower stability of the systems with this ff. A time-step of 2 fs was used for the simulations with the LIPID14 + ff14SB combination and for one of the two replica simulations of CYP 2C9 and CYP 2C19 with the GAFF + ff99SB combination. The other simulations with the GAFF + ff99SB combination were run with a time-step of 1.5 fs to ensure reasonable bilayer stability in simulations exceeding 100 ns.

The electrostatic interactions were calculated using the PME method. All bonds to hydrogen atoms were constrained with the SHAKE algorithm^67^. Temperature was controlled by Langevin dynamics with a damping coefficient of 0.5 ps^−1^ at 310 K on non-hydrogen atoms. Pressure was controlled by the Nosé-Hoover Langevin piston method with oscillation and damping times of 1000 fs. The GAFF-LIPID force-field requires simulations to be done under constant surface tension (NPγT ensemble) to maintain the structural properties of the membrane bilayer^46^ and thus for production runs (without harmonic restraints), a surface tension of 60 dyn/cm in the x-y plane was used. The LIPID14 parameters, on the other hand, have been optimized for use with anisotropic pressure coupling (NPT ensemble) on the basis of tests for pure membrane systems without any protein present^37^. Therefore, anisotropic pressure coupling was used for simulations of our protein-membrane systems with LIPID14.

### Definitions of the parameters characterizing the position of the protein with respect to the membrane

The position and orientation of the protein with respect to the membrane was quantified by calculating various angles and distances, as previously reported^16–18^. Three vectors were defined as follows (see Fig. 1): v1, from the center of mass (CoM) of the backbone particles (CG representation) or atoms (AA representation) of the first 4 residues to the CoM of the last 4 residues of the I-helix; v2, from the CoM of the first 4 residues of the C-helix to the CoM of the last 4 residues of the F-helix; v3, the vector between the CoMs of the first and last four residues of the TM- helix, and the z-axis, which is perpendicular to the membrane. The angle α was defined as the angle between v1 and the z-axis, and angle β was defined as the angle between v2 and the z-axis. Thus, angles α and β together define the orientation of the CYP globular domain with respect to the membrane normal. The heme tilt angle, defined as the angle between the heme plane (defined by the four nitrogen atoms coordinating the iron) and the z-axis, was also monitored for direct comparison with experimental measurements. The axial distance of the CoM of the globular domain to the CoM of the lipid bilayer was also monitored during the trajectories. The TM-helix tilt angle or angle γ in the lipid membrane was defined as the angle between v3 and the z-axis.

### Analysis of all-atom molecular dynamics simulations

The VTMC tool, which combines both Voronoi tessellation and Monte Carlo methods to assign a Voronoi polygon to each lipid, was used to compute area per lipid (APL)^68^. The method computes the APL for boundary lipids (lipids within 5 Å of the protein) and non-boundary lipids (other lipids) (script available at: https://github.com/prajwal07/Calculate_LIPID_parameters_using_Voronoi_Method). The electron density profile across the membrane bilayer was computed from the atomic distribution on the membrane normal using the ccptraj module^69^ of AmberTools (ambermd.org). The membrane was divided into slabs of 0.1 Å thickness in the z-dimension. The calculated electron density in the slab was then divided by the average cross-sectional area to obtain the electron density in e/Å^3^. The electron density was computed and time averaged for the last approximately 66 ns and 76 ns for MD simulations of the CYP 2C9 systems with LIPID14 + ff14SB ff and GAFF-LIPID + ff99SB ff, respectively. Carbon-hydrogen (deuterium) order parameters were computed using the VMD plugin MEMBPLUGIN^70^. The protein secondary structure profiles^64^ were computed using the cpptraj module^69^ of the Amber14 software package (ambermd.org).

The molecular graphics representation in Figs. 1 and 2 was generated using VMD 1.9 (www.ks.uiuc.edu/Research/vmd/)^20^. Plots for Figs. 3, 4A–C and S1, 2 were generated using Xmgrace^38^. Fig. 4D was generated using g_lomepro^50^. Supplementary Figs. S3–6 were generated using Gnuplot^71^.

## Supplementary information

Supplementary Information.

Supplementary Data.

## Data Availability

The trajectories and associated files generated in the current study are available from the corresponding author upon reasonable request.
