# Supplementary material for: The Effect of Force-Field Parameters on Cytochrome P450-Membrane Interactions: Structure and Dynamics
Source: Sci Rep. 2020 Apr 29;10:7284. doi: 10.1038/s41598-020-64129-7 (PMC7190701; doi:10.1038/s41598-020-64129-7)
Supplement: Supplementary file 1 — Supplementary Information. [file 41598_2020_64129_MOESM1_ESM.pdf]

SUPPORTING INFORMATION for:

The Effect of Force-Field Parameters on Cytochrome P450-Membrane Interactions: Structure and Dynamics

Ghulam Mustafa,[a+] *Prajwal P. Nandekar,*[a,b+] *Goutam Mukherjee,* [a,b] *Neil J. Bruce,*[a] *Rebecca C. Wade*[a,b,c,*]

[a] Molecular and Cellular Modeling Group, Heidelberg Institute for Theoretical Studies (HITS), Heidelberg, Germany
[b] Zentrum für Molekulare Biologie der Universität Heidelberg, DKFZ-ZMBH Alliance, INF 282, 69120 Heidelberg, Germany
[c] Interdisciplinary Center for Scientific Computing (IWR), Heidelberg University, INF 368, 69120 Heidelberg, Germany

Contents:

Supplementary Figures S1-S6

Supplementary Data: Six coordinate files in PDB format. These contain the coordinates of the three protein-membrane systems from the final snapshots from simulations with the two force field combinations as specified in the file names.

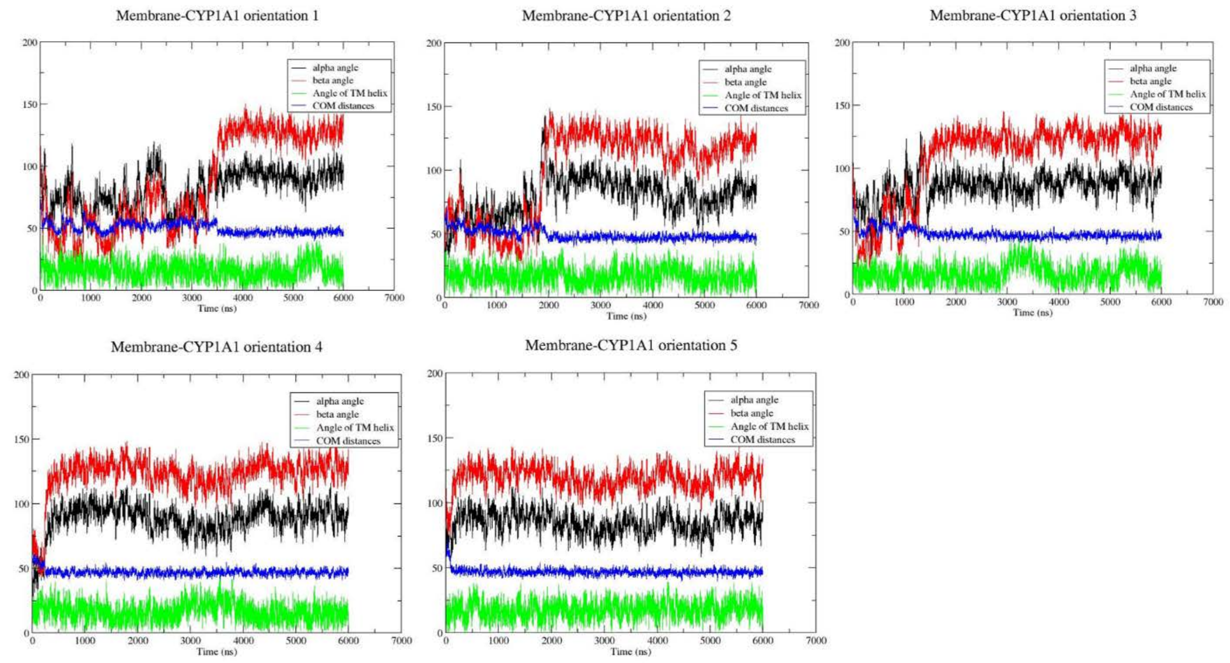

Figure S1. Convergence of the CYP 1A1 protein-POPC bilayer system during five 6-microsecond coarse-grained (CG) simulations starting from different initial conformations of the flexible linker. The evolution of the parameters defining the orientation and insertion depth of the globular domain and the angle of the transmembrane (TM) helix with simulation time is plotted. In all five simulations, the globular domain converged to the same orientation and depth in the bilayer. Similarly, convergence to a single orientation was observed in the CG simulations run for CYP 2C9 and CYP 2C19. The plots were generated using Xmgrace (plasma-gate.weizmann.ac.il/Grace/).[1]

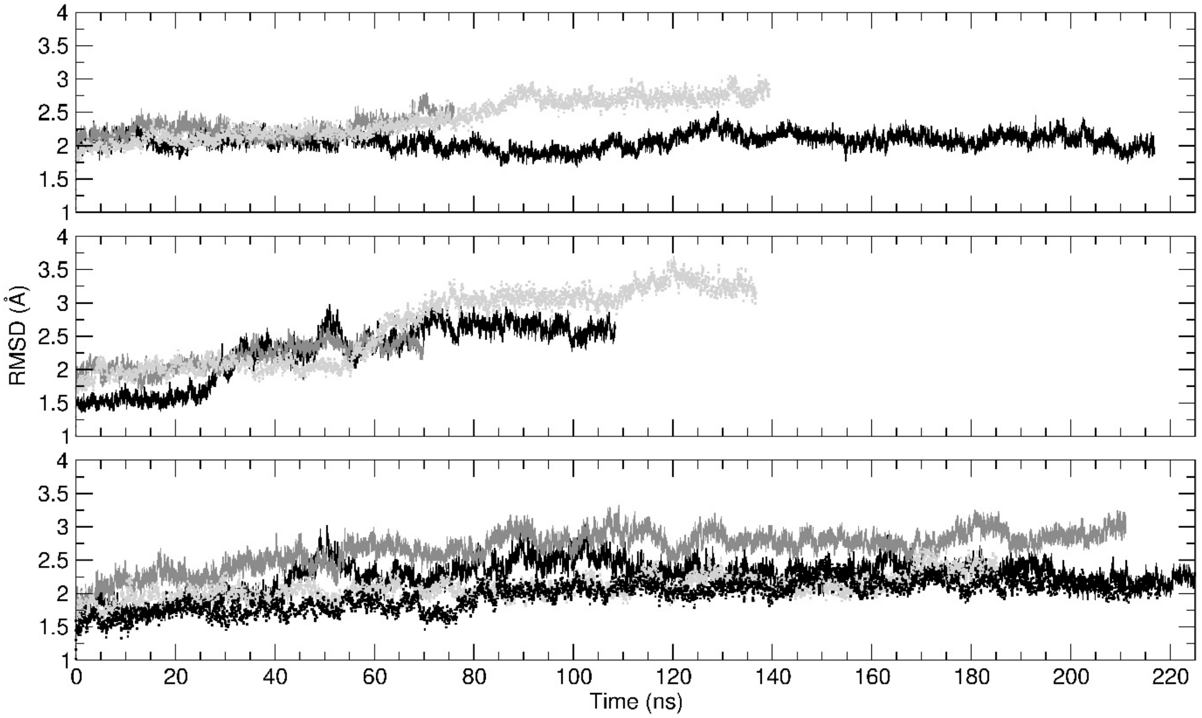

Figure S2. Plots of the evolution of protein backbone Cα atom root mean squared deviation (RMSD) of the CYP globular domain with respect to the energy minimized structure along the AA MD simulations of CYP 2C9 (top), CYP 2C19 (middle), and CYP 1A1 (bottom) with LIPID14+ff14SB (black; the two replicas for CYP 1A1 are shown in solid and dotted lines) and GAFF+ff99SB (light and medium gray; 2 replica simulations for each system). The plots were generated using Xmgrace (plasma-gate.weizmann.ac.il/Grace/).[1]

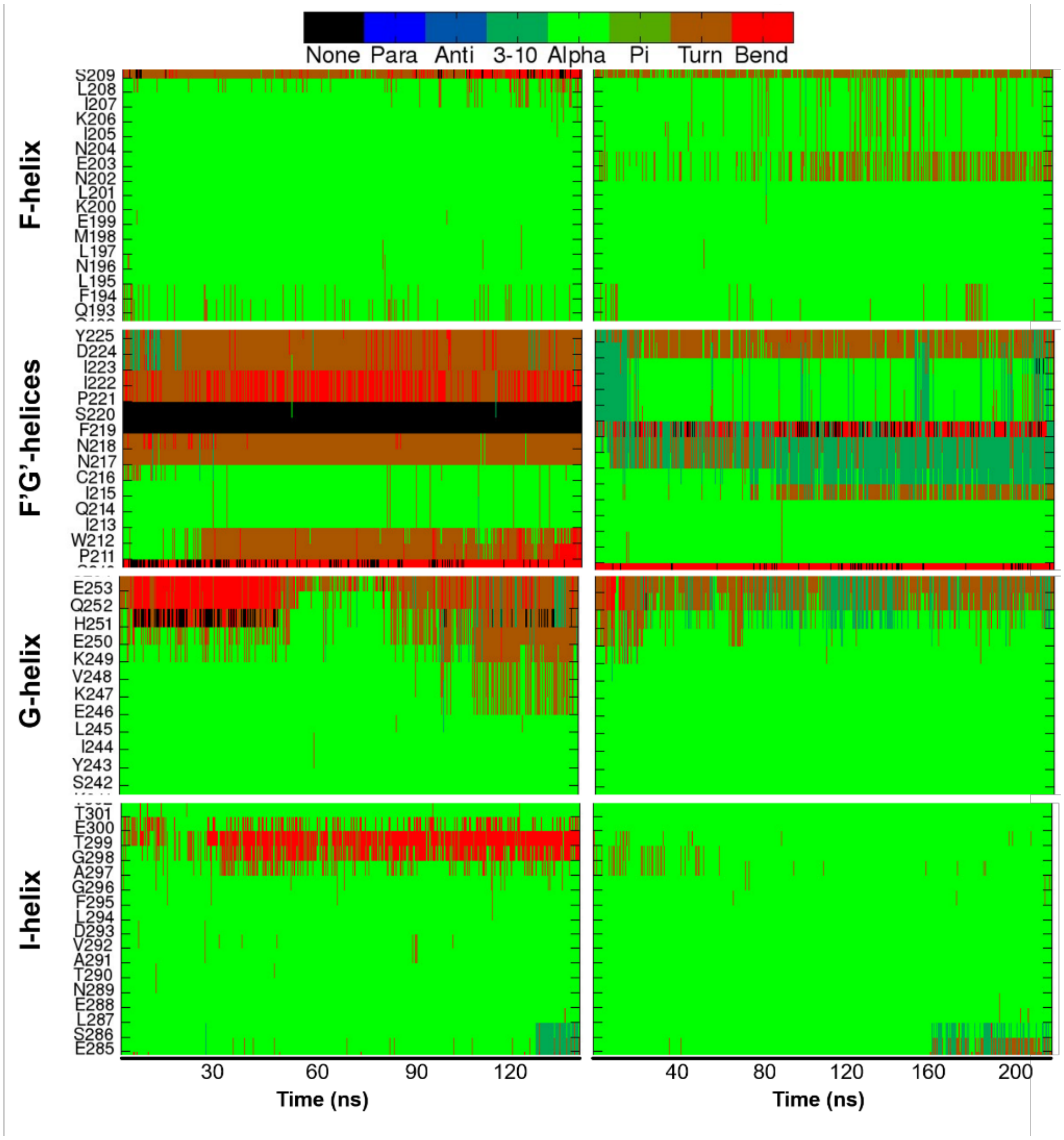

Figure S3. Evolution of the secondary structure during MD simulations of the CYP 2C9-membrane system with two force-fields, GAFF-LIPID+ff99SB (139.4 ns, left panels) vs LIPID14+ff14SB (216.9 ns, right panels) shown for the F helix, F'G' helices, G helix and I helix regions (from top to bottom). The color bar denotes the different secondary structures[2]. For the simulations with GAFF-LIPID+ff99SB, secondary structure distortion or bending of helices was observed in the F' helix (residues 211-213 and 217-218) and G' helix (residues 221-225) and in the G helix (residues 246-250); unwinding of the central I helix (residues 298-300) was also seen. The figure was generated using Gnuplot v. 5 (www.gnuplot.info/).[3]

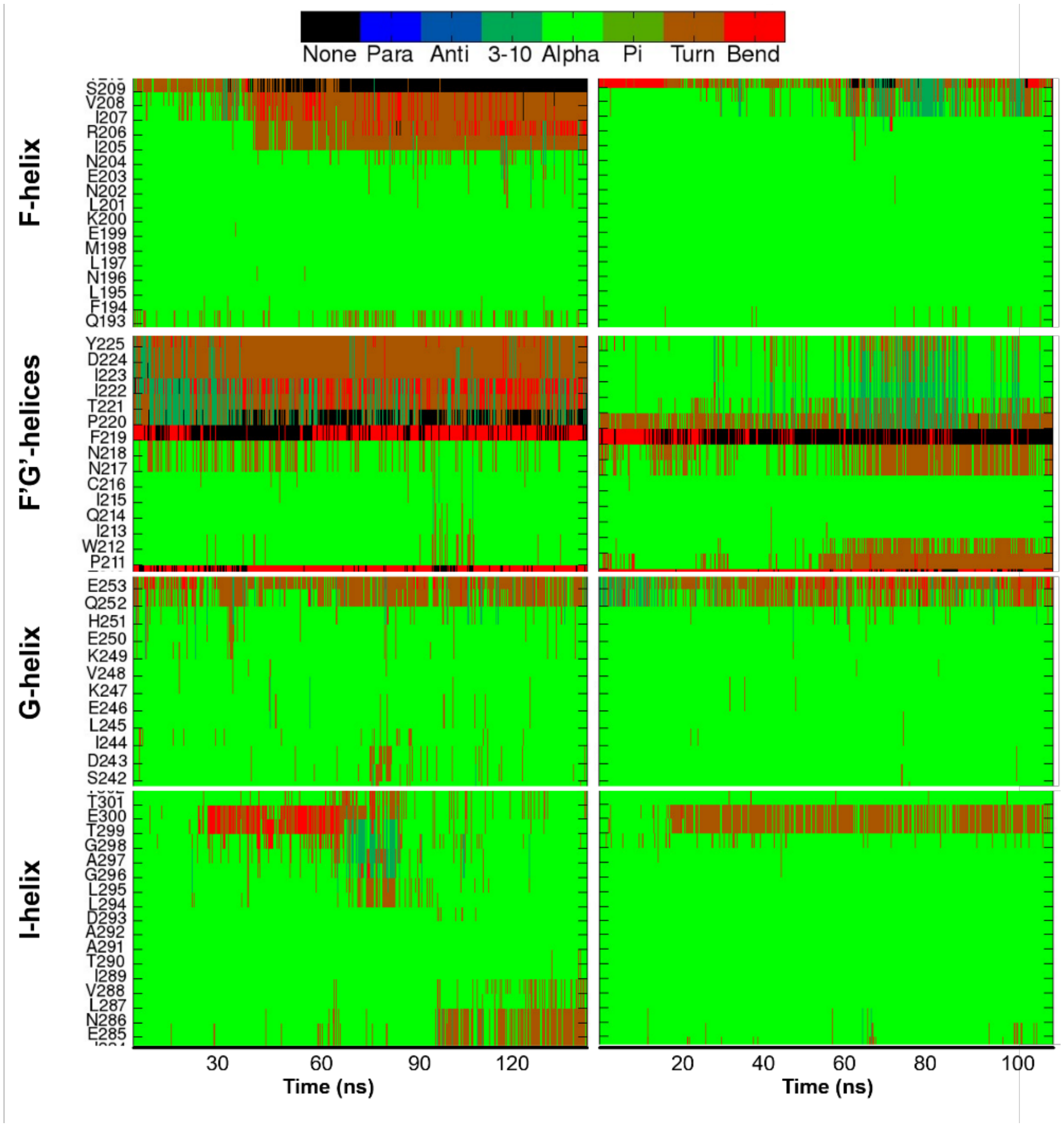

Figure S4. Evolution of the secondary structure during MD simulations of the CYP 2C19-membrane system with two force-fields, GAFF-LIPID +ff99SB (136.7 ns, left panels) vs LIPID14+ff14SB (108.4 ns, right panels) shown for the F helix, F'G' helices, G helix and I helix regions (from top to bottom). The color bar denotes the different secondary structures[2]. Distortion in the terminal F helix residues 205-208, the G' helix and unwinding in the I helix were observed in CYP 2C19 when simulated with GAFF-LIPID +ff99SB. The figure was generated using Gnuplot v. 5 (www.gnuplot.info/).[3]

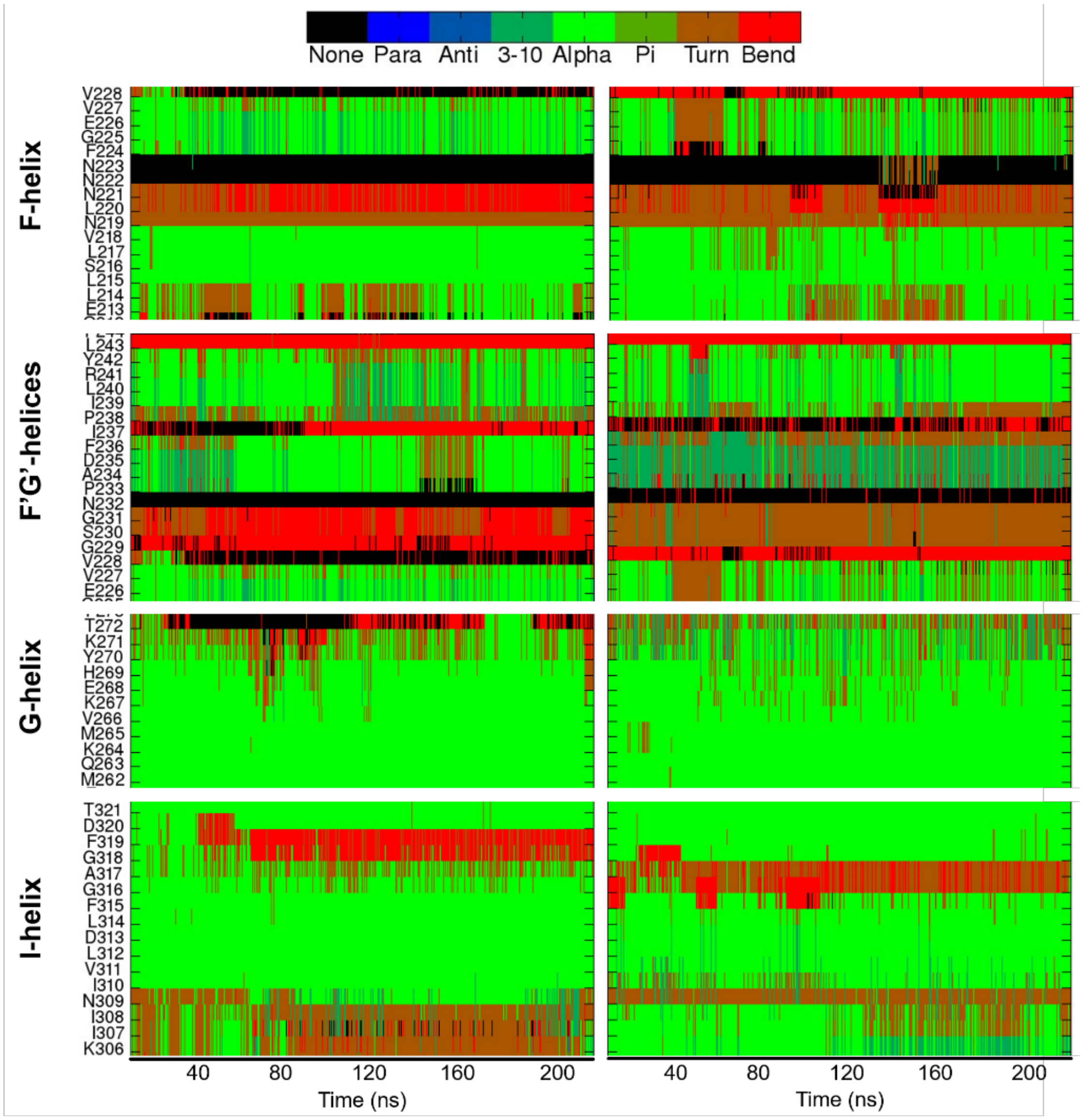

Figure S5. Evolution of the secondary structure during MD simulations of the CYP 1A1-membrane system with two force-fields, GAFF-LIPID +ff99SB (211.2 ns, left panels) vs LIPID14+ff14SB (225.2 ns, right panels) shown for the F helix, F'G' helices, G helix and I helix regions (from top to bottom). The color bar denotes the different secondary structures[2]. The secondary structure behaves similarly in the simulations for the two force fields. The figure was generated using Gnuplot v. 5 (www.gnuplot.info/).[3]

(A)

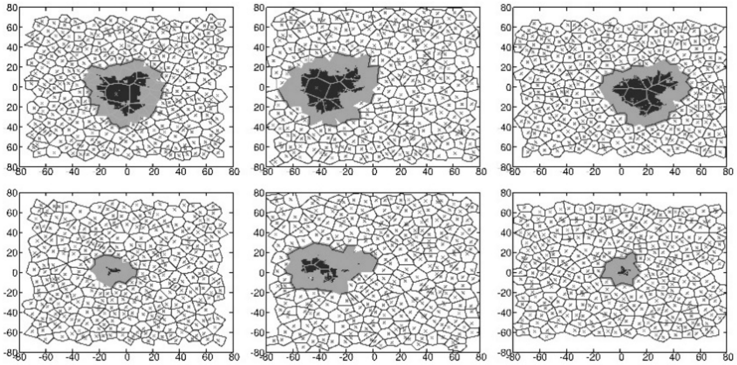

(B)

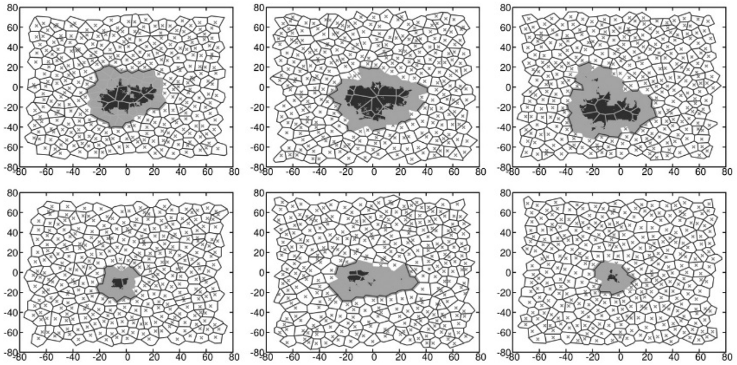

(C)

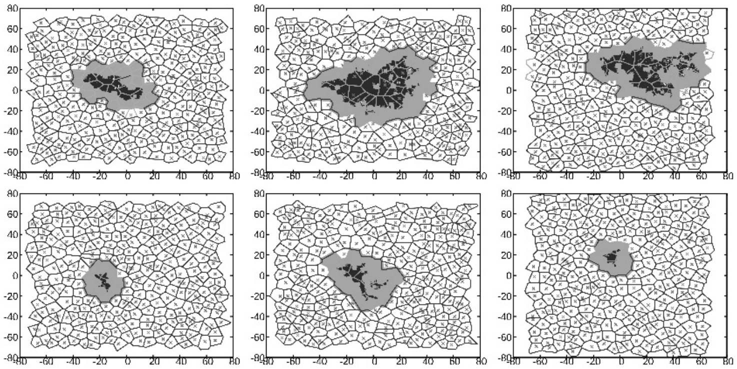

Figure S6. The area per lipid (APL) calculated for the (A) CYP 2C9, (B) CYP 2C19 and (C) CYP 1A1 systems by Voronoi tessellation for non-boundary and Monte Carlo integration for boundary phospholipids: initial frame (left) and last frame for GAFF-LIPID+ff99SB (middle, (after simulation of 76 ns for CYP 2C9, 69.7 ns for CYP 2C19 and 211.2 ns for CYP 1A1)) and LIPID14+ff14SB (right, (after simulation of 216.9 ns for CYP 2C9, 108.4 ns for CYP 2C19 and 225 ns for CYP 1A1)) for the upper (upper panel) and lower (lower panel) layers. The black regions show protein atoms. The boundary (gray) and non-boundary (white) phospholipids are represented by polygons. + marks show the center of mass of each non-boundary phospholipid molecule. Distances along the x and y axes are given in Å. Changes in the shape of the bilayer and the immersion of the protein in the bilayer during the simulations and differences between the CYP systems are discernable. The figure was generated using Gnuplot v. 5 (www.gnuplot.info/).[3]

REFERENCES:

(1) Turner, P. J. XMGRACE, Version 5.1. 19. *Cent. Coast. Land-Margin Res. Oregon Grad. Inst. Sci. Technol. Beaverton, OR* (2005).

(2) Kabsch, W. & Sander, C. Dictionary of protein secondary structure: pattern recognition of hydrogen-bonded and geometrical features. *Biopolym. Orig. Res. Biomol.* **22**, 2577–2637 (1983).

(3) Racine, J. gnuplot 4.0: a portable interactive plotting utility. *J. Appl. Econom.* **21**, 133–141 (2006)
